# A Peptide–Lectin Fusion Strategy for Developing a Glycan Probe for Use in Various Assay Formats

**DOI:** 10.3390/chemosensors7040055

**Published:** 2019-11-13

**Authors:** Butaek Lim, LeNaiya Kydd, Justyn Jaworski

**Affiliations:** Department of Bioengineering, University of Texas at Arlington, Arlington, TX 76010, USA

**Keywords:** oligomannose, lectin, *Pseudomonas fluorescens agglutinin*, agglutination assay, Western blot assay

## Abstract

While nucleic acid and protein analysis approaches continue to see significant breakthroughs, analytical strategies for glycan determination have by comparison seen slower technological advances. Here we provide a strategy for glycan probe development using an engineered lectin fusion that can be incorporated into various common pathology lab assay formats including Western blot and agglutination assays. In this proof of concept, we use the natural lectin, *Pseudomonas fluorescens agglutinin* (PFA), capable of binding core Man alpha(1-3)-Man alpha(1-6)-Man units, where this lectin has previously been shown to bind to the glycans presented by the gp120 coat protein of (HIV) Human Immunodeficiency Virus. In our strategy, we engineered the lectin to possess a fusion of the biotin mimetic tag equence of amino acids V-S-H-P-Q-A-P-F. With the glycan receptive PFA directly linked to the biotin mimic, we could facilitate a probe for various standard clinical assay formats by virtue of coupling to streptavidin-HRP (horseradish peroxidase) or streptavidin beads for Western blot and agglutination assays respectively. We found the PFA fusion retained low nanomolar affinity for gp120 by ELISA (Enzyme Linked Immunosorbent Assay) and microscale thermophoresis. This probe engineering strategy proved effective in the relevant assay formats that may now allow detection for the presence of glycans containing the core Man alpha(1-3)-Man alpha(1-6)-Man units recognized by PFA.

## 1. Introduction

Glycans have traditionally been complicated to study as their structural complexity allowing sugar building blocks to be linked at different sites and with different stereochemistries goes beyond the relatively simple linear template-driven synthesis of nucleic acids and proteins [1]. While the difficulty in their analysis, detection, and even synthesis continues, the key roles of glycans have become apparent within the full spectrum of biological processes and are a distinct requirement for life [2-4]. Tools for genetic and proteomic level testing are abundant but we have yet to possess sufficient glycan-related diagnostic tools that may be integrated into standard practice. Because of this bias in attention, there remains a lot to be understood regarding glycans in terms of not only basic science but also from the perspective of clinical diagnostics and is thus deserving of special consideration [5]. The objective of the following study is to assess a biotin mimetic peptide fusion to a well-known lectin as a proof-of-concept oligosaccharide probe that may be utilized with a variety of common clinical assay formats. Traditional glycan analysis techniques include chromatographic analysis of metabolically radio-labeled glycans, as well as capture with lectin affinity chromatography, and glycosidase treatment for chemical analysis of the constituent sugars [6-8]. Other techniques have included the use of sequential exoglycosidase digestion coupled with methylation analysis utilizing chromatography, mass spectroscopy, and ^1^H NMR [9-11]. Current analytical strategies for examining protein glycosylation include enrichment techniques prior to mass spectroscopy methods, where lectin chromatography [12,13] as well as chemical techniques, including the use of boronic acid [14] and hydrazide chemistry [15], have been applied to enrich glycoproteins. Enzymatic removal can facilitate the isolation of the glycans alone, which can have even more difficulty in separation as glycans have poor adsorption to reverse phase high performance liquid chromatography (HPLC) matrices. Glycan separation by HPLC thus requires derivatization by reductive amination [16], but this can be a source of artifacts [17]. A range of other options for glycan (either free or labeled) separation include capillary electrophoresis, tandem anion-exchange, porous graphitic carbon liquid chromatography, and hydrophilic interaction chromatography [4]. Assuming effective separation, analysis of which glycan structures are present requires the examination of mass spectral data or chromatographic/electromigratory assessment of the retention times given a definitive set of reference glycans. Since these aforementioned techniques are time consuming approaches, require multiple steps, and necessitate a high level of expertise, there have been recent groundbreaking works to simplify glycan analysis which have made the use of lectin-based arrays [18]. Lectins are natural glycan receptors that exist with specific recognition for a number of different glycan structures, and the use of lectin-based arrays as sensing modalities have been found to expedite the examination of protein glycosylation to under 6 h [19]. Still, the number of possible combinations of glycans which exist far outweigh the number of currently known lectins for specific glycan structures, and as such lectin arrays must utilize classification techniques based on “fingerprinting” of glycan interactions with an array containing lectins with relatively broad specificities. An example includes an array of 24 plant lectins with overlapping specificities [18]. While individual lectins may have some promiscuity in their binding [20], the fact that an individual glycan can provide a unique fingerprint across the array still holds value in the analysis. Growing research efforts to identify and examine new lectins with distinct specificities as well as novel assay approaches utilizing lectins is still in demand, and with that we were motivated to pursue this research.

**Figure 1.**
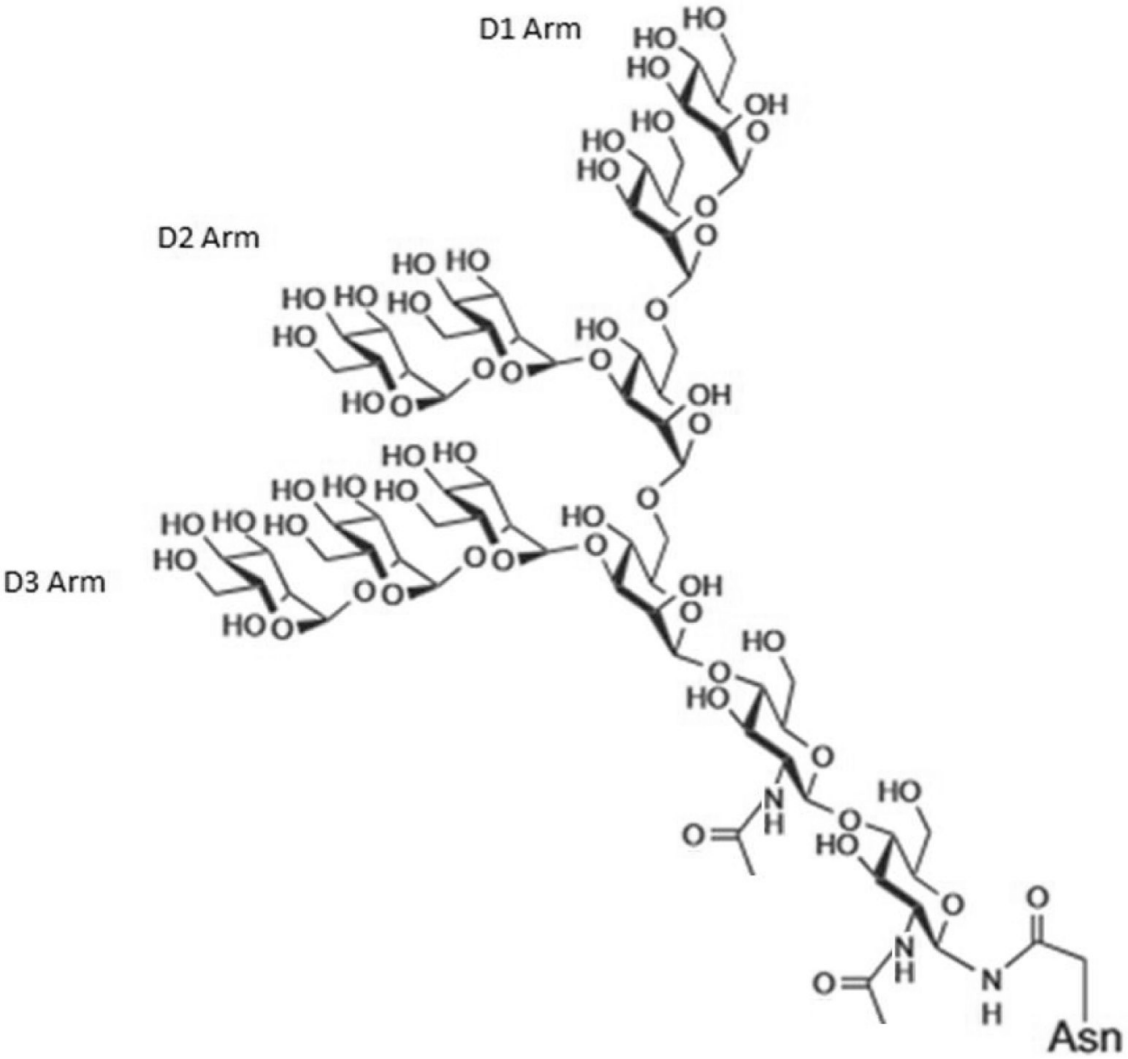
Structure of an example high-mannose-type glycan (Man_9_, one of the known glycans displayed by HIV coat protein gp120) possessing alpha 1,2 linkages between linear mannose units at the D1, D2, and D3 arm and possessing alpha 1,3 and alpha 1,6 linkages at branch points.

Serum level changes in glycan profiles as well as isolation of glycoproteins with irregular glycan structures have proven to be a distinguishing characteristic between cancer vs. control patients for a range of malignancies [21]. One form of irregular glycosylation type is high-mannose-type glycans displayed on viruses (one such glycan, Man_9_, is shown in Figure 1), including HIV and rotavirus [22], but also on the immunoglobulin of certain B cell lymphomas [23]. In addition to being displayed on subsets of B cell lymphomas, high-mannose-type glycans represent an interesting glycan-based biomarker found on a number of other cancer cells including the surface of human lung adenocarcinoma, colorectal cancer, as well as on the epidermal growth factor receptor of epidermoid carcinoma and gastric cancer cell lines [24]. In the context of healthy mammalian systems, mannose units are not a typical terminating moiety but rather are masked by other sugar moieties capped onto the end of mannose units after trimming [25]. This trimming of natural high mannose occurs as Man_9_ is formed briefly and cleaved to Man_5_ by mannosidases within the ER/early Golgi serving as the precursor internal core of more mature glycans [26]. Aberrant display of high mannose along the secretory pathway is handled by intracellular human lectins (like OS-9) that traffic such glycoproteins for proteasomal degradation by the ERAD (endoplasmic reticulum associated degradation) pathway [27]. Extracellular human C-type lectins, such as DC-SIGN (dendritic cell specific intercellular adhesion molecule-3 grabbing non-integrin) and MBL (mannose binding lectin), can bind aberrant oligomannosylation, requiring a calcium ion for coordination with the glycan, to facilitate their removal [28]. It has been reported that monosaccharide fucose units can also coordinate with the calcium ion in DC-SIGN and even manifest interaction with α1–α2 linked fucose as presented by histo blood group antigens, highlighting a prime example of the cross-specificity or promiscuity that exists in many lectins [29,30]. There are also several plant and microbe derived lectins that recognize oligomannose units in a calcium independent manner including *Pseudomonas fluorescens agglutinin* (PFA), which is a member of the *Oscillatoria agardhii agglutinin* homologue family of lectins capable of recognizing the Man alpha(1-3)-Man alpha(1-6)-Man core [31] present on high mannose and hybrid type glycans. Because PFA lectin has been found to recognize this core branch point, it provides broader selectivity for Man_6_ to Man_9_ variants as compared to other lectins [32,33]. This reported promiscuity for Man_5_GlcNAc_2_, Man_6_GlcNAc_2_, Man_7_GlcNAc_2_, Man_8_GlcNAc_2_, and Man_9_GlcNAc_2_ highlights that a single lectin may not be sufficient to distinguish glycan structure. Because these structural variants are all displayed by the HIV coat protein gp120 [34], the PFA lectins ability to bind the Man alpha(1-3)-Man alpha(1-6)-Man core has recently been exploited for inactivation of HIV transmission but has yet to be used as a sensing moiety [35].

**Figure 2.**
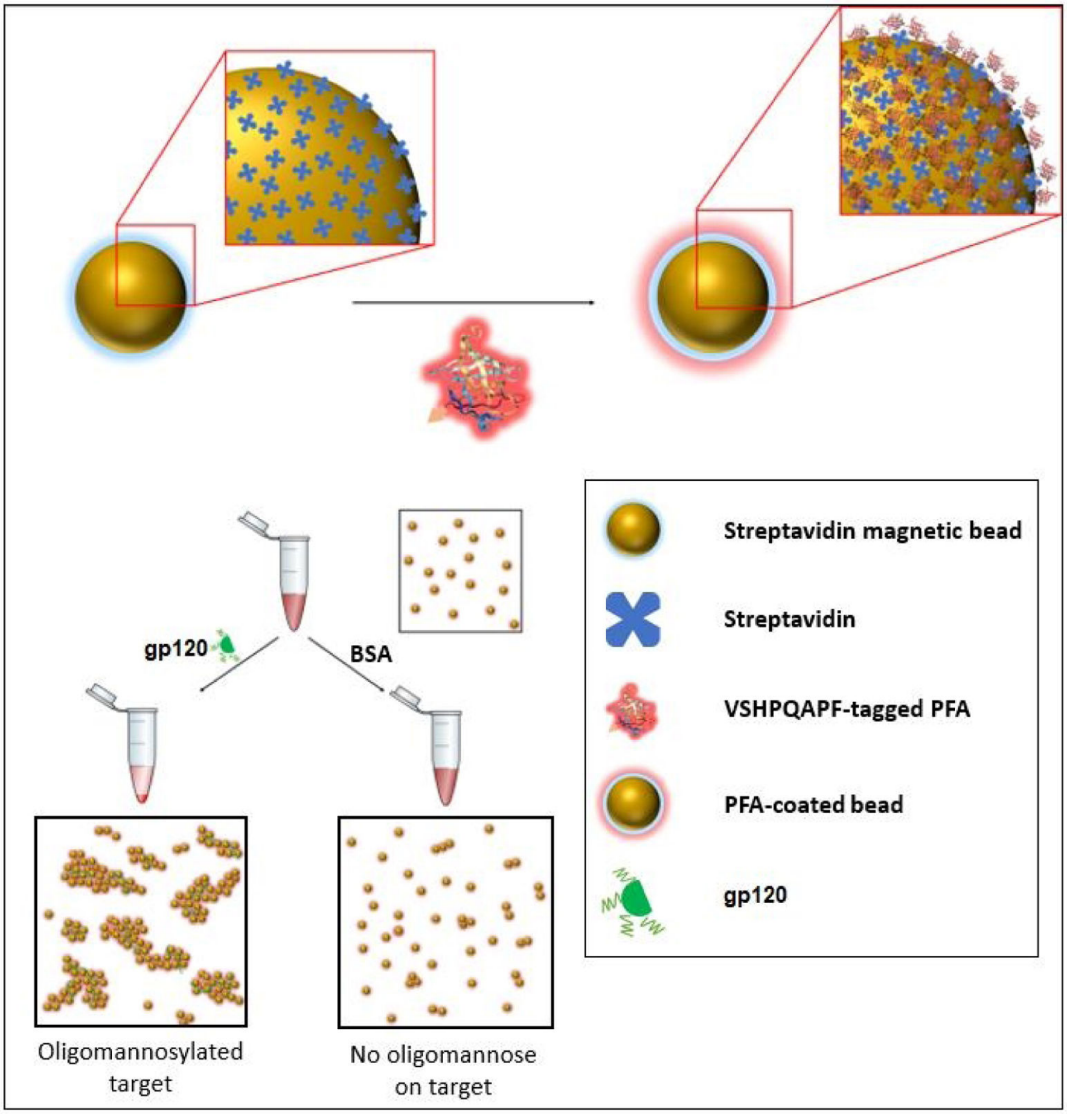
Schematic of one of the sensing modalities (specifically an agglutination assay) in which the PFA engineered with a biotin mimetic fusion peptide of the sequence VSHPQAPF may be implemented to provide detection of the presence of oligomannosylation.

In this work, we examine a strategy of glycan probe development using biomimetic peptide–lectin fusion approach which in this proof-of-concept utilized *Pseudomonas fluorescens agglutinin* (PFA) for the assessment of glycans in different assay formats. We have engineered the PFA lectin to possess a biotin mimetic tag (VSHPQAPF) [36-38] given the ability of the bacterial derived PFA to be easily expressed in a bacterial system to afford a glycan probe that could be implemented in different detection assay modalities. The PFA lectin exhibits two binding sites for the Man alpha(1-3)-Man alpha(1-6)-Man core on opposing ends of its beta-barrel-like architecture. The engineered PFA fusion to the biotin mimetic peptide proved useful in providing a means for determining the presence of glycans by fluorometric and enzymatic reporting mechanisms in our assays as well as affording the mechanism by which to carry out a bead-based agglutination assay as highlighted in Figure 2. This serves as a starting point for future work in developing a simple and reliable strategy for detecting the presence of other glycans by implementing different lectin-based receptive moieties and further encourages us to develop a future set of engineered lectins with varying specificities to be used in a form of glycan analysis toolkit.

## 2. Materials and Methods

### 2.1. Materials and Validation of Key Biological Reagents

To provide the source of materials in this study, gBlocks were obtained from Integrated DNA Technologies for cloning of the PFA fusion protein. The oligomannose bearing gp120 was purchased from Sigma Aldrich and confirmed for the presence of oligomannose using known commercial probes capable of binding to oligomannose (Supplementary Material Figure S1), specifically the anti-oligomannose antibody 2G12 obtained from Polymun and the Man alpha(1-3)-Man binding lectin GNA (*galanthus nivalis agglutinin*) obtained from Vector Laboratories as well as fluorescein labeled GNA from the same source. In these validation assays, glycosidase treatment with Endo H (endoglycosidase H obtained from New England Biolabs) was used to remove oligomannose from glycoproteins by cleaving the chitobiose (GlcNAc2) core to confirm the specificity of the 2G12 and GNA for oligomannose. DC-SIGN/Fc (dendritic cell-specific intercellular adhesion molecule-3 grabbing non-integrin fused to fragment crystallizable domain) and DC-SIGNR/Fc (DC-SIGN related protein fused to fragment crystallizable domain) were purchased from R&D Systems. Unless otherwise specified, all reagents used for cloning were obtained from New England Biolabs and all reagents used for expression, purification, and analysis were obtained from Sigma Aldrich.

### 2.2. Expression of PFA

For production of the engineered PFA lectin, a pET14b bacterial expression vectors was used. A custom double stranded DNA gBlock possessing the open reading from the PFA gene was purchase from Integrated DNA Technologies. The gBlock underwent PCR with the following primers to produce restriction sites of BamHI and NdeI for cloning into the pET14b expression vector: For PFA (CTAAACATATGATGTCCAAATACGCGGTCGCAAATCAATG); Rev PFA (GATTGGTTTTCGCGGACAGATTGAGTAGGGATCCATAACA). Generation of the engineering VSHPQAPF fusion to PFA was carried out by using the following primers: BiotinMim1 (CTGTGGGTGCGAGACCTCAATCTGTCCGCGAAAACC) and BiotinMim2 (ATACGGGATCCCTAAAAAGGTGCCTGTGGGTGCGAG). After confirmation of the clones and expression testing for IPTG inducible expression within the *E. coli* BL21 DE3 expression system, larger scale 1 L cultures were grown at room temperature in LB media proceeded until an optical density of 0.3 at which point cultures were placed at 16 °C for 30 min, followed by induction with IPTG (isopropyl thiogalacto pyranoside) and overnight growth at 16 °C. After collection of the cell pellet, lysis buffer was added followed by rocking incubation for 30 min on ice and then, 10 min of probe sonication on ice (programmed at 1 s on and 1 s off at 60% power in 1 min intervals). The lysate was centrifuged at 16,000 rpm for 1 h and the supernatant was filtered prior to undergoing nickel affinity chromatography at 1 mL/min flow rate at 4 °C. After binding to the Ni column, the sample was washed for 1 h and then subjected to an increasing gradient of imidazole for release from the column. The eluted fractions were assessed by SDS PAGE (sodium dodecyl sulfate polyacrylamide gel electrophoresis) and fractions containing the pure protein of interest were pooled and dialyzed into the appropriate storage buffer.

### 2.3. Fluorescence Labeling of PFA

Amine-reactive protein labeling kit (NT-647-NHS) was used for PFA labeling. Labeling buffer was prepared by adding 3 mL distilled water to labeling buffer salt (included in the kit). Zeba spin columns (included in the kit) were prepared according to manufacturer’s protocol. Briefly, the bottom cap was removed, and upper cap was loosened followed by centrifugation at 1500 g for 1 min to remove storage liquid. Column was equilibrated by washing with 300 μL of labeling buffer three times. Then, 125 μL of PFA solution was placed and buffer exchanged PFA was collected in a microcentrifuge tube after centrifugation at 1500 g for 2 min. NT-647 dye was reconstituted by adding 30 μL of 100% DMSO (dimethyl sulfoxide) yielding approximately 470 μM and diluted further to achieve 11 μM. This concentration is about three times more than that of the PFA protein that was to be labeled (3.88 μM). Using 250 μL of PFA and NT-647 dye, each were mixed in a 1:1 volume ratio and incubated for 30 min at room temperature in the dark. During that time, a PD minitrap desalting column was prepared according to the manufacturer’s manual. After equilibrating the column three times with PBS (1X phosphate buffered solution), 500 μL of labeling reaction was loaded to the center of desalting column followed by the addition of 600 μL of PBS. Finally, 100 to 150 μL fractions of the eluate were collected and measured for their concentration by Bradford assay where the final PFA-NT-647 concentration was 11 μg/mL, which was equivalent to 392 nM.

### 2.4. Competitive Binding Assays

A total of 100 μL of 1 mg/L gp120 was absorbed to 96-well immunoplates overnight at 4 °C and each well was washed with 300 μL of PBS once. The plate surface was blocked with 1% BSA (bovine serum albumin) in PBS (phosphate buffered solution) for 2 h at room temperature with horizontal shaking at 50 rpm. After discarding the blocking solution, 100 μL of 400 nM PFA was added to the wells and incubate for 10 min with shaking. After PFA binding, 100 μL of 40 nM biotinylated GNA was directly added to the wells and incubated for 1 h then each well was washed with 300 μL of washing buffer (1% BSA in 1X PBST (phosphate buffer solution containing tween-20). Then, 100 μL of 1 μg/mL streptavidin-HRP in washing buffer was added to each well and incubated for 30 min. After washing unbound streptavidin-HRP with 300 μL of washing buffer, 100 μL of TMB solution was added and the signal was recorded at 450 nm using an Epoch2 microplate spectrophotometer (BioTek Inc., Winooski, VT, USA) after 2 min development and stopping the TMB-peroxide reaction by adding 100 μL of 0.16 M sulfuric acid. To examine if PFA was binding to oligomannose, we carried out an experiment to see if the PFA would inhibit the GNA binding. This experiment was conducted as above but with the addition of 100 uL of 400 nM PFA (non-biotinylated) to the wells prior to addition of the biotinylated GNA. After confirming the ability of PFA to bind to the glycans of gp120, we utilized soluble mannose in another competitive binding assay wherein 100 μL of 1 mg/L gp120 was absorbed overnight to a 96-well immunoplate, incubated at 4 °C, and each well was washed with 300 μL of PBS once. Again, the plate surface was blocked with 1% BSA in PBS for 2 h at room temperature with horizontal shaking at 50 rpm. After discarding the blocking solution, 100 μL of 400 nM PFA was added to the wells, along with 0–2000 mM soluble mannose directly added to the wells. After shaking and incubating for 1 h, each well was washed with 300 μL of washing buffer (1% BSA in PBST) and again ELISA was carried out with 100 μL of TMB solution to observe the remaining PFA with the signal recorded at 450 nm using the Epoch2 microplate spectrophotometer (BioTek Inc.) after 10 min development with the TMB-peroxide reaction was stopped by adding 100 μL of 0.16 M sulfuric acid.

### 2.5. SDS PAGE and Western Blot

Proteins to be separated were treated as follows: 3 μL sample loading buffer (containing SDS, glycerol, Tris-HCl, β-mercaptoethanol, and bromophenol blue) was added to 9 μL of protein and the mixture was heated for 10 min at 95 °C to be denatured. The 12%/4% separating/stacking gel was made for electrophoresis. Samples were loaded onto the gel and run using SDS-PAGE running buffer (containing SDS and Tris-HCl, pH 8.3) for the first 30 min at 80 V, then an additional 1 h at 120 V until maximally resolved. Separated protein was transferred to the nitrocellulose membrane using a Bio-Rad mini-trans blot system as follows: nitrocellulose membrane was presoaked in 1X transfer buffer (containing Tris-HCl, Glycine and methanol, pH 8.3) after assembling a blot sandwich, followed by wet blotting for 16 h at 4 °C. The membrane was then blocked in 1% BSA 1X PBS overnight at 4 °C in a humid chamber. For staining, to identify the ability of PFA to serve in affinity to oligomannosylated proteins, we first conducted incubation of 12 μL of 10 μM PFA-HPQ (PFA bearing the VSHPQAPF fusion) with 4 μL of 1 mg/mL strep-HRP for 30 min at room temperature in 4 mL of washing buffer. These mixtures were then directly added to the blocked membrane for 1 h at room temperature with shaking, followed by washing three times with washing buffer (containing 1% BSA and 0.5% Tween-20 in 1X PBS). The membrane was developed in 2 mL of 1X AEC solution (0.02% 3-amino-9-ethylcarbazole in N, N Dimethylformamide, 0.015% hydrogen peroxide in acetate buffer pH 5.5) for 20 min. An identical staining technique was used for DC-SIGNR/Fc and DC-SIGN/Fc primary using HRP conjugated anti-IgH as secondary. For the Western blot conducted in Supplementary Figure S2, samples of gp120 were added with or without prior glycosidase treatment. EndoH and a1-2,3,6 Mannosidase obtained from New England Biolabs were used for the gp120 samples in the right and left lane, respectively, using the manufactures recommended reaction conditions. After transfer to nitrocellulose membrane and blocking with BSA, staining was carried out as described above but using a 1:1 premix of strep-HRP and PFA-HPQ.

### 2.6. Bead-Based Agglutination Assay

A total of 30 μL of 4 mg/mL streptavidin beads (streptavidin covalently coupled with <1 μm super paramagnetic particles) was suspended in 1 mL of binding buffer (20 mM Tris-HCl, 0.5 M NaCl, 1 mM EDTA pH 7.5). Coating of the beads with our engineered PFA bearing the VSHPQAPF fusion (termed PFA-HPQ for short as the HPQ motif is the widely found consensus region of streptavidin binder) was carried out with the addition of 5 μL of 435 μg/mL PFA-HPQ protein, followed by shaking at 80 rpm for 1 h at room temperature. Beads were removed and washed with 1 mL of binding buffer three times using magnetic capture for isolation during washing. The beads were then blocked with 1 mL of blocking buffer (1% BSA in PBS) for 1 h. The beads were then diluted with two volumes of washing buffer (0.5% Tween 20,1% BSA, 1X PBS). Then, 100 μL of diluted beads were used for each group either with or without the incorporation of PFA-HPQ. After adding an additional 5 μL of either 100 μg/mL of gp120 as the oligomannosylated target or BSA as the target without oligomannose. Microscopy images (acquired using a Nikon Ti-2) of the samples were captured at zero minutes, 40 min, and 80 min to assess the extent of agglutination.

## 3. Results

### 3.1. Expression and Confirmation of Engineered PFA

**Figure 3.**
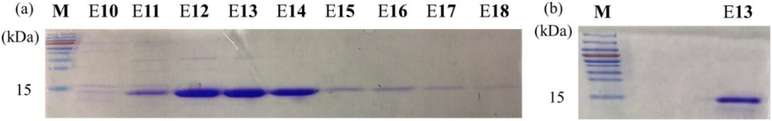
SDS-PAGE of protein ladder marker, M, and PFA fractions (eluents E10 through E18) after affinity chromatography (**a**) before and (**b**) after dialysis showing pure PFA protein isolated with expected molecular weight of 14 kDa.

**Figure 4.**
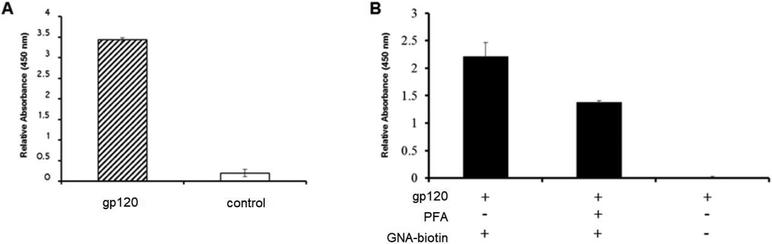
ELISA signals showing binding of (**A**) GNA (*galanthus nivalis agglutinin*) lectin to immobilized gp120 target and (**B**) competitive elution of GNA lectin when in the presence of PFA lectin.

From two 1 L cultures of *E. coli* BL21 DE3 harboring the pET14b expression vector, we obtained 8 fractions of different purity of the PFA, where the purest fractions E13 and E14 were combined and used for our studies yielding 14 mL of 435 μg/mL of PFA as determined by Bradford assay. Having purified the lectin *Pseudomonas fluorescens agglutinin* (Figure 3), we initially examined its ability to bind to the glycans of gp120 as compared to GNA (*Galanthus nivalis agglutinin*) which is known to bind glycans with (α-1,3)-linked mannose residues. We determined the GNA was capable of binding to the glycans presented by the HIV glycoprotein gp120, which we then used as one of two glycan bearing targets in this study (Figure 4A). A competitive binding assay was initially used to examine the ability of our PFA product to bind the glycans displayed on gp120. From Figure 4B, we can see that by pre-incubating the gp120 with PFA, we could significantly reduce the binding ability of the GNA which provided an indication that PFA could bind and mask a portion of glycan sites of the gp120. While we were not interested in confirming the exact specificity of the lectins given the likelihood that GNA and PFA could have occupied different sites, (despite reports that these lectins both recognize the mannose branch point), we merely used the initial results to justify moving forward to conducted further binding assessment of the PFA.

### 3.2. Examining Binding Affinity of Engineered PFA

**Figure 5.**
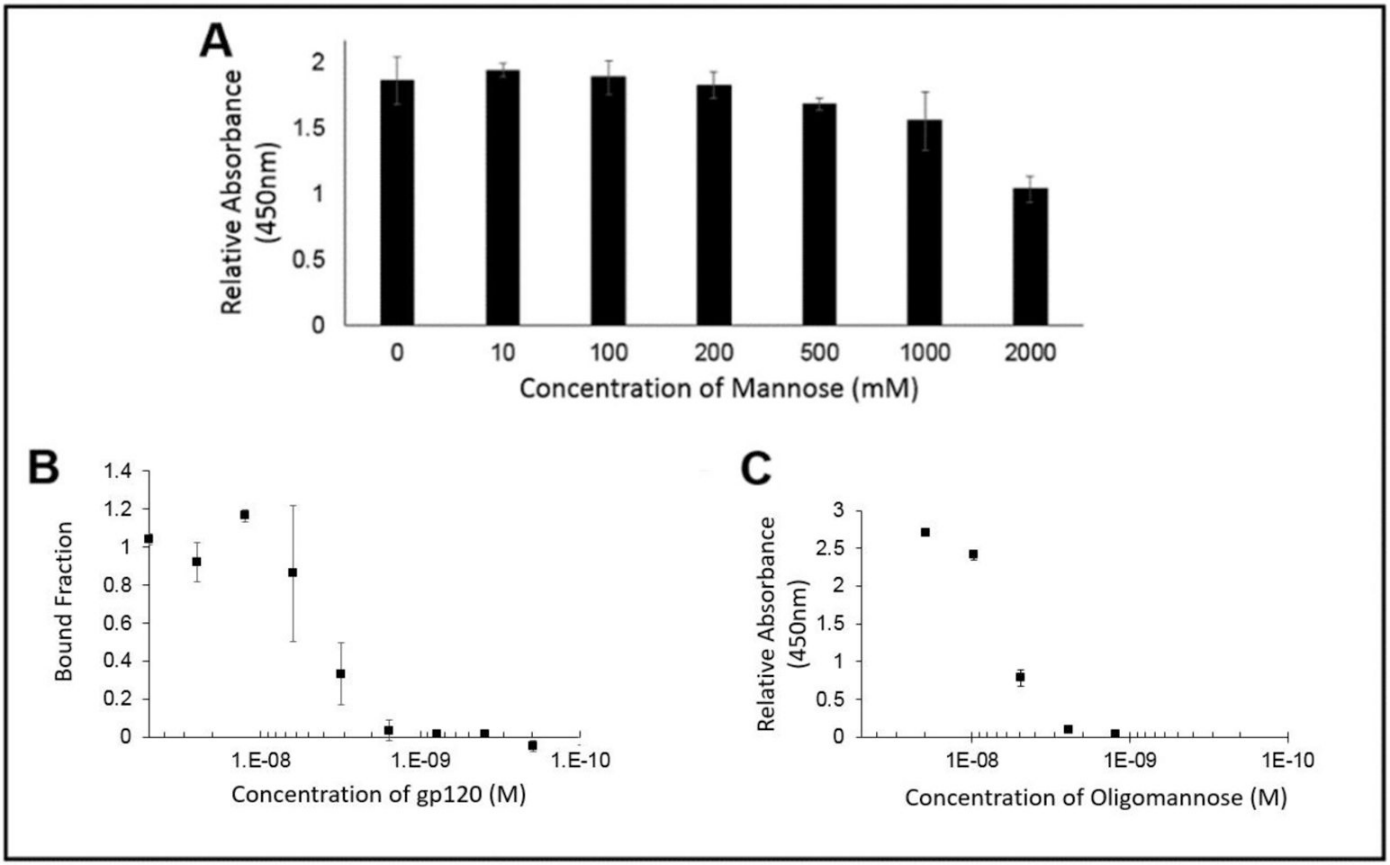
(**A**) ELISA signal of PFA against an immobilized gp120 target in the presence of increasing amount of soluble mannose showed no substantial inhibition in PFA binding until greater than 200 mM of soluble mannose was added; (**B**) Microscale thermophoresis and (**C**) ELISA of PFA against gp120 target showing similar binding constants, K_d_ ~4 nM.

In order to demonstrate if the PFA ability to recognize the glycans was displayed by gp120, wells coated with gp120 were exposed to PFA in the presence of increasing concentrations of soluble mannose. As seen if Figure 5A, when labeling the remaining PFA with an enzymatic probe, the colorimetric signal remained consistent up to the condition of nearly 200 mM of free mannose. From this assessment of the binding ability of PFA to the glycans of gp120, we see that there was no significant inhibition by mannose in preventing PFA binding to oligomannose for physiologically relevant levels of added mannose, which supports that PFA may hold value as a diagnostic tool in biologically relevant fluids where free mannose would not exist beyond the 200 mM concentration needed to begin inhibit binding of PFA to the glycans of gp120.

In order to provide direct evidence for the ability of PFA to bind to glycans possessing the Man alpha(1-3)-Man alpha(1-6)-Man core unit, we utilized the technique of microscale thermophoresis for quantitative assessment of binding affinity. Having prepared the fluorescently labeled PFA possessing the NT-647 fluorophore and confirmed its labeling efficiency, we setup a series of experiments to first identify the relative extent of binding ability of PFA for gp120 to properly choose the dilution range for quantitative examination of the binding affinity of PFA for the Man alpha(1-3)-Man alpha(d-6)-Man bearing target. Using the NT-647 labeled PFA samples with or without gp120 in 0.1%) PBST, we could see a clear shift in the thermophoretic mobility and thus, conducted a full serial dilution of the gp120 ligand concentration as shown in Figure 5B. The measured decrease in the bound fraction with decreasing amounts of gp120 revealed an effective binding affinity of 4.1 ± 1.4 nM. To validate these results, we utilized a conventional ELISA approach in which another Man alpha(1-3)-Man alpha(1-6)-Man glycan unit displaying protein target (soy bean agglutinin conjugated to HRP) was exposed to serial dilutions of PFA to identify via the colorimetric signal generated by TMB the binding signal against PFA, as shown in Figure 5C. Examining the PFA for its binding affinity to oligomannose by ELISA and microscale thermophoresis confirmed a similar value of approximately 4 nM. Having validated the binding capability of PFA for Man alpha(1-3)-Man alpha(1-6)-Man displaying glycans, we then examined how modification of an engineered PFA to display the biotin-mimetic HPQ tag could make this receptor adaptable to common sensor modalities such that we may detect the presence of oligomannose as shown in the following section.

### 3.3. Bead Assay and Western Blot Assays

**Figure 6.**
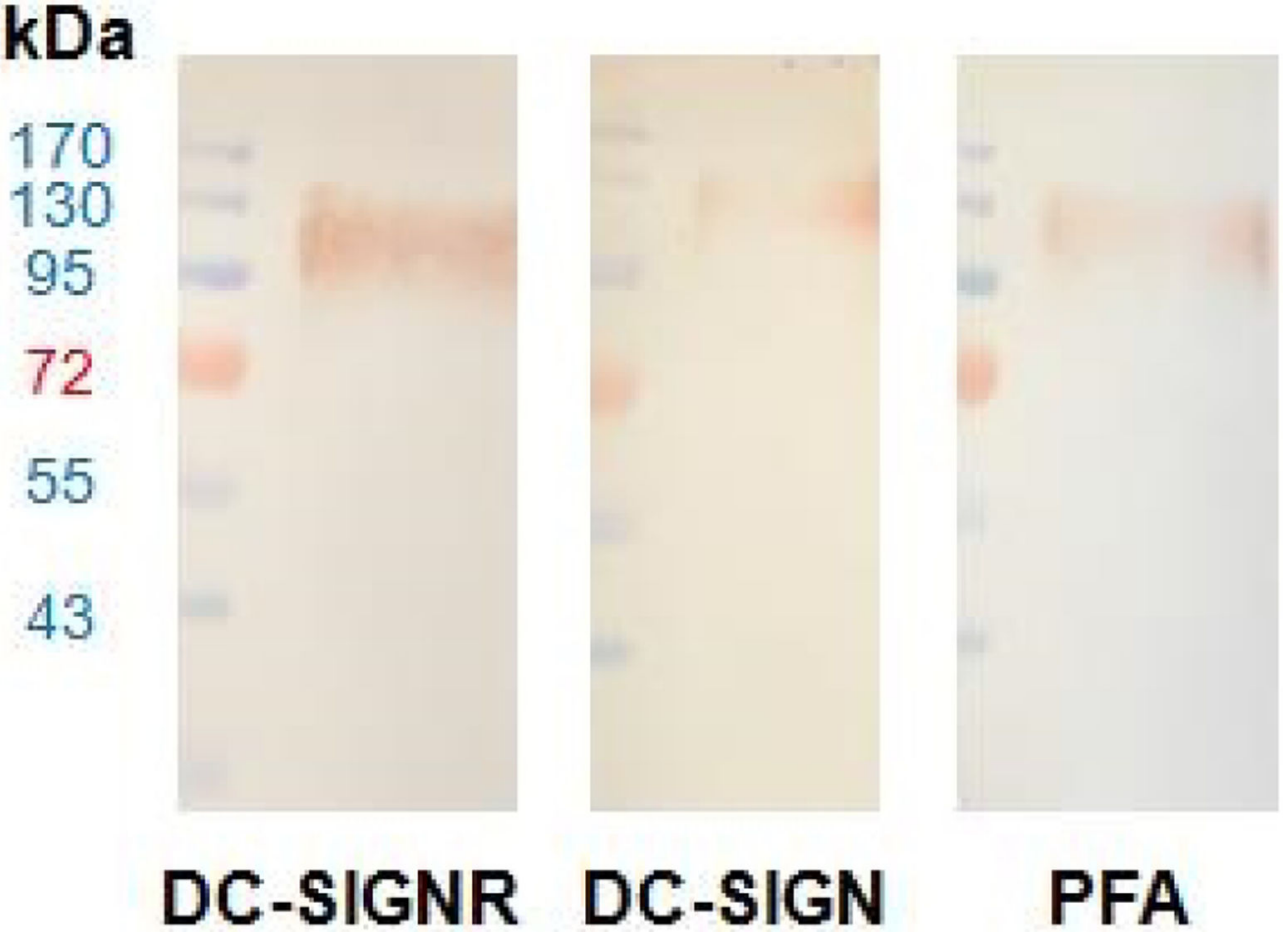
Western blot with ladder in lane 1 and glycan displaying gp120 in lane 2 utilizing primary receptors of DC-SIGNR/Fc (**left**), DC-SIGN/Fc (**middle**), and PFA-HPQ (**right**). Secondary incubation was carried out with HRP conjugated anti-IgH for DC-SIGNR and DC-SIGN, while the use of Strep-HRP was used for PFA. The presence of gp120 target could be identified using the experimental PFA-HPQ to the same extent as that of the positive controls of DC-SIGNR and DC-SIGN, which are natural target receptors for HIV transfection.

After assessing the binding affinity of PFA, we utilized the engineered PFA fusion which bears the biotin mimetic tag VSHPQAPF known to have high affinity to streptavidin in order to facilitate its implementation with standard pathology lab analysis techniques including Western blot and agglutination assays. In conducting these experiments, we utilized the gp120 envelope glycoprotein of HIV that displays glycans that natively bind to DC-SIGN and DC-SIGNR for infection of dendritic cells and endothelial cell, respectively [39]. As positive controls for our Western blot experiments, we thus utilized DC-SIGN and DC-SIGNR as natural receptors for the glycans displayed by gp120 to compare our engineered PFA-based probe. From Figure 6, of gp120 transferred to a nitrocellulose membrane, we can clearly see the AEC stained signal using PFA-HPQ (PFA bearing the biotin mimetic VSHPQAPF tag) primary and strep-HRP (streptavidin conjugated to horseradish peroxidase) secondary. In fact, the PFA-HPQ signal against gp120 was comparable to that of the positive control signals determined for DC-SIGN and DC-SIGNR.

**Figure 7.**
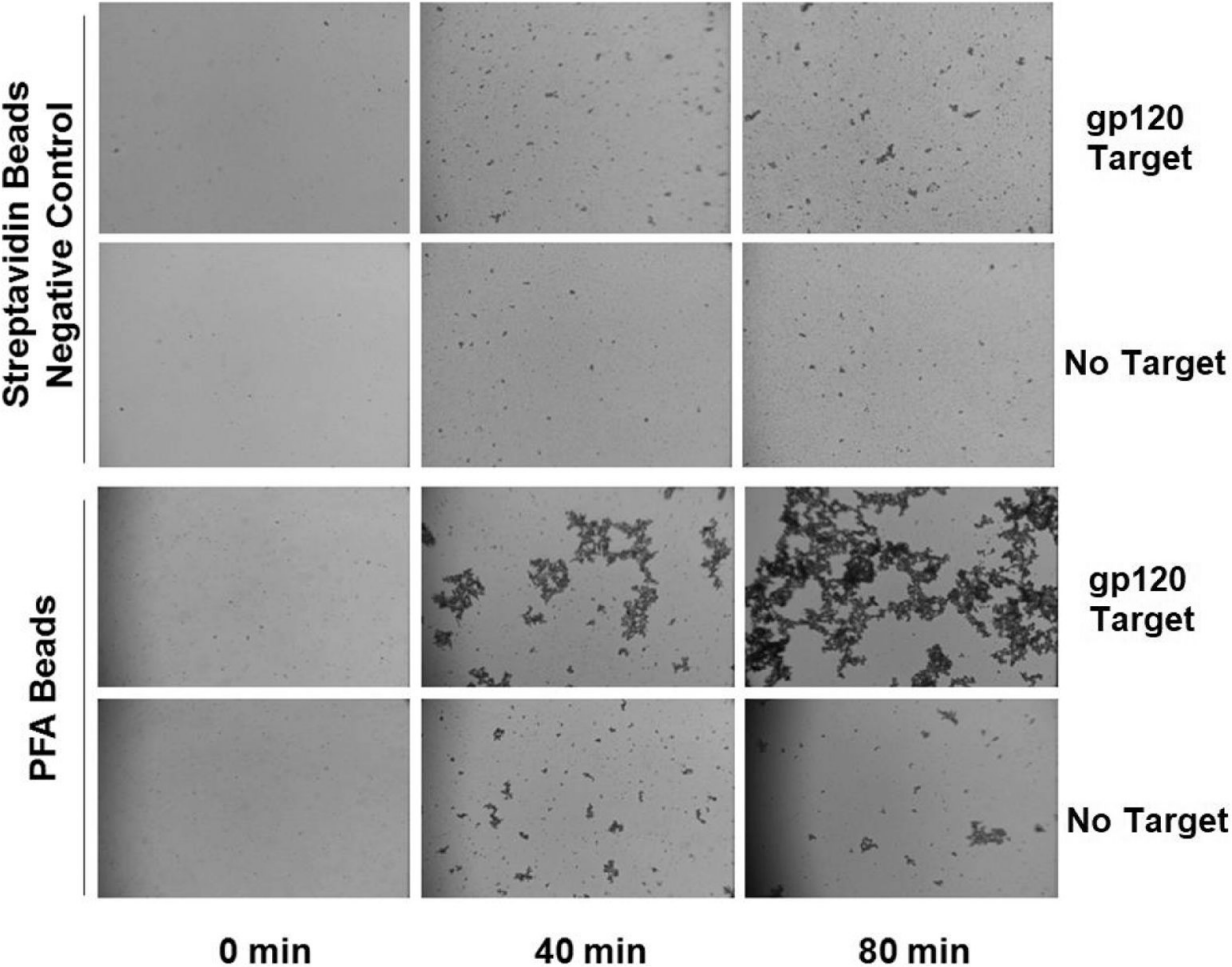
Agglutination assay for gp120 using PFA-HPQ labeled streptavidin beads as compared to unlabeled streptavidin beads, showing clear increasing agglutination response of PFA-HPQ beads to gp120 target as a function of time.

The engineered PFA-HPQ fusion did well in the common Western blot assay format for recognizing the presence of glycans displayed on the gp120. To examine the PFA-HPQ performance with another typical detection modality, we carried out immobilization of the PFA-HPQ onto streptavidin beads (<1 μm diameter super paramagnetic iron oxide particles) in order to determine its adaptability to agglutination assays. In the course of conducting the agglutination assays against glycosylated gp120 target and control for both the streptavidin bead as well as the PFA-HPQ coated streptavidin bead, we find the onset of a distinct agglutination event for the PFA-HPQ coated beads when exposed to the gp120 target. This qualitative binding signal occurs with increasing relative aggregation compared to the control samples as time progresses. This format provides an easy to understand assay for the presence or absence of glycans such as in this case glycans possessing Man alpha(1-3)-Man alpha(1-6)-Man core units as was shown here in Figure 7 for the case of gp120. Having assessed the binding affinity of PFA and incorporating the VSHPQAPF biotin mimetic tag, the engineered PFA has shown to offer a useful component for a variety of assay formats to detect oligomannosylation.

## 4. Discussion

The objective of this study was to show a biotin mimetic peptide fusion to a glycan receptive moiety that could provide an oligosaccharide probe development strategy that could be useful to various assay formats relevant to clinical pathology labs. We selected the lectin PFA as our glycan receptive moiety based on reports of its high yield in bacterial expression along with the extensive literature reporting the ability of PFA to bind the glycans of the HIV coat protein gp120 which we could use as a model target for our proof of concept study [32,33,35]. While endogenous human lectins, DC-SIGN and DC-SIGNR, are known to be capable of recognizing oligomannose, we examined a bacterial derived lectin that may be produced in high yield in our bacterial expression system. Indeed, we find that expression proceeded well in our *E. coli* BL21 DE3 system, given the nature of the protein and after purification by nickel affinity chromatography afforded approximately 6 mg of pure protein from 2 L of culture. Before proceeding with examining the PFA, we first confirmed our assays using positive controls of GNA lectin, DC-SIGN, and DC-SIGNR used throughout this study. To assure that our receptive lectin moiety was indeed undergoing glycan binding, we conducted competitive binding experiments, Western blots of gp120 under various glycosidase treatments (Figure S2), as well as quantitative binding assays. GNA is reported to recognize the Man alpha(1-3)-Man unit and through lectin frontier database [40], the interaction graph reveals GNA to bind to those such glycans which possess (α-1,3)-linked mannose residues with high affinity for Man_3_ [40]. We hence utilized it as our competitive binder and in doing so found that PFA reduced the extent of GNA binding to gp120 by virtue of masking a proportion of the glycan binding site. This offered an initial promising result that our engineered PFA could bind to glycans presented by gp120 and we assessed that free mannose up to 200 mM had no inhibitory effect on the PFA binding. To provide assessment of the glycan binding capability of the engineered PFA, we carried out two distinct binding affinity assays to cross-validate our quantitative assessment. We confirmed, by both ELISA and microscale thermophoresis, a similar glycan binding affinity of the PFA lectin to be approximately 4 nM.

The strong glycan binding affinity seen for the engineered PFA could be considered sufficient for a receptive moiety for in vitro diagnostic applications, but to adopt this receptor for common clinical workflows we needed to engineer the PFA for both Western blot and agglutination assays. We have previously used the well-known VSHPQAPF peptide as a biotin mimetic motif capable of high affinity binding to streptavidin [36]. Because of the wide commercial availability of streptavidin conjugated products, including fluorescent molecules, horseradish peroxidase (HRP), and magnetic beads for clinical assays, the engineered PFA-HPQ fusion can now be utilized in a variety of detection platforms. Here, we have incorporated the VSHPQAPF as a fusion onto the PFA to specifically facilitate its implementation with strep-HRP secondary reporters for Western blot, as well as for capture onto coatings of streptavidin beads for use in agglutination assays. Specifically utilizing the glycan bearing target gp120 as the analyte for detection, we see that the PFA-HPQ receptor conjugated with strep-HRP performed well as a glycan probe in providing a visually detectable signal by Western blot. In fact, this signal was comparable to that of natural receptors for the gp120 glycans, namely DC-SIGN and DC-SIGNR, which are receptors present on dendritic cells and endothelial cells that act as virion binding sites during HIV transfection.

In looking at an agglutination assay platform, typical manifestations utilize latex beads coated with antigen and are used to identify the presence of specific antibodies, or conversely coated with antibodies and used to identify the presence of antigens. In this demonstration, we utilized streptavidin coated magnetic beads having a diameter of less than one micron to serve as the solid phase component. As these particles are super paramagnetic iron oxide, they will not aggregate unless exposed to an external magnetic field of sufficient strength which allowed us to easily wash, collect, and re-suspend the beads during coating with the engineered PFA-HPQ lectin. The success of our probe in this agglutination assay to detect glycans of gp120 possessing the Man alpha(1-3)-Man alpha(1-6)-Man core unit in a sample was confirmed using these PFA-HPQ coated streptavidin beads, where the positive signal became apparent at the 40 min time point post-introduction of the glycan bearing gp120 sample, and to a much greater extent at the 80 min time point. With this proof-of-concept aggregation confirmed for the PFA coated beads in the presence of glycan bearing gp120 target, we show that our strategy of using an engineered lectin fusion is successful in providing a detectable signal for future development or potential incorporation with existing agglutination assay platforms. Different formats of agglutination assays have been used commercially and in literature to generate a variety of visual feedbacks to the user as a result of the aggregation of the bead sample and can even provide, in some cases, a color change or optical signal to confirm a positive result in the sample [41,42]. The importance of this work in showing that this proof-of-concept engineered lectin fusion may be incorporated into a variety of detection modalities highlights the possibility of this serving as a robust strategy, and we hope to continue to develop a series of related lectin-based receptors for other glycans that may provide a toolkit/panel to begin assessing the vast repertoires of glycosylation motifs of clinical interest.

## Supplementary Material

Supplementary Material Figure S1: Validation of gp120 display of oligomannose and binding of GNA to oligomannose by (A) western blot of gp120 with and without EndoH glycosidase treatment using the anti-oligomannose antibody (2G12) for staining or (B) using the lectin GNA for staining. (C) Microscale thermophoresis (MST) experiments using gp120 (208.5 nM) and fluorescein labeled GNA (20 nM) were carried confirm by the MST traces showing a distinct shift in the thermophoretic mobility of the GNA alone (blue), as compared to GNA with addition of gp120 (green) (inset showing the shift in normalized fluorescence based on the readings taken in the red column (hot) and blue column (cold) time frame representing after and before onset of the thermal gradient)
Figure S2: Additional experiment of PFA fusion probe staining against gp120 with and without glycosidase treatment. Western blot of gp120 that had undergone Mannosidase treatment (left), gp120 without glycosidase treatment (middle), and gp120 that had undergone Endo H treatment (right).
