# Supplementary material for: A Peptide–Lectin Fusion Strategy for Developing a Glycan Probe for Use in Various Assay Formats
Source: Chemosensors (Basel). Author manuscript; Available in PMC 2020 Aug 12. (PMC7423246; doi:10.3390/chemosensors7040055)
Supplement: Supplementary Material [file NIHMS1066841-supplement-Supplementary_Material.pdf]

chemosensors

Supplementary Material

A Peptide–Lectin Fusion Strategy for Developing a Glycan Probe for Use in Various Assay Formats

Butaek Lim, LeNaiya Kydd and Justyn Jaworski *

Department of Bioengineering, University of Texas at Arlington, Arlington, TX 76010, USA; butaek.lim@mavs.uta.edu (B.L.); lenaiya.kydd@mavs.uta.edu (L.K.)

* Correspondence: Justyn.Jaworski@uta.edu

Received: 30 September 2019; Accepted: 8 November 2019; Published: date

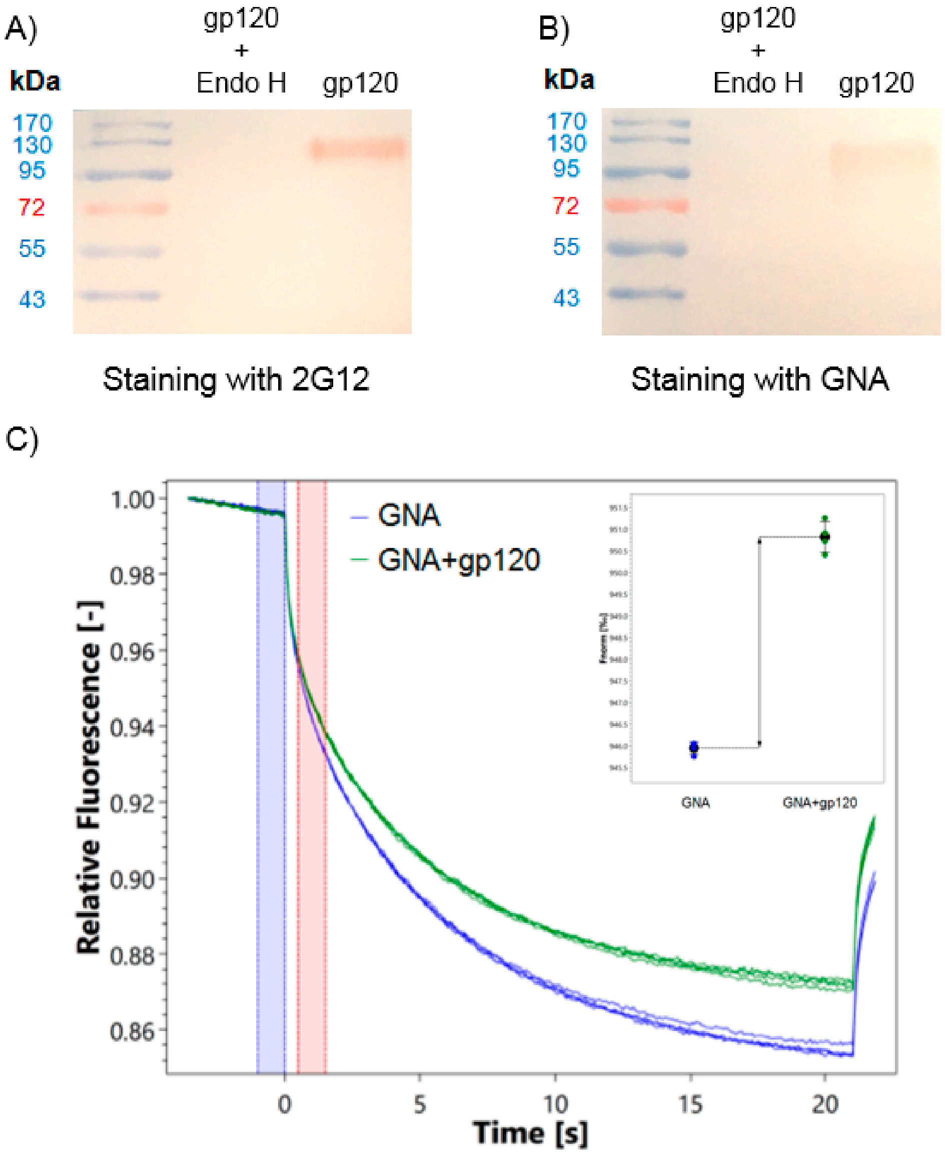

Figure S1. Validation of gp120 display of oligomannose and binding of GNA to oligomannose by (**A**) western blot of gp120 with and without EndoH glycosidase treatment using the anti-oligomannose antibody (2G12) for staining or (**B**) using the lectin GNA for staining. (**C**) Microscale thermophoresis (MST) experiments using gp120 (208.5 nM) and fluorescein labeled GNA (20 nM) were carried confirm by the MST traces showing a distinct shift in the thermophoretic mobility of the

GNA alone (blue) as compared to GNA with addition of gp120 (green) (inset showing the shift in normalized fluorescence based on the readings taken in the red column (hot) and blue column (cold) time frame representing after and before onset of the thermal gradient).

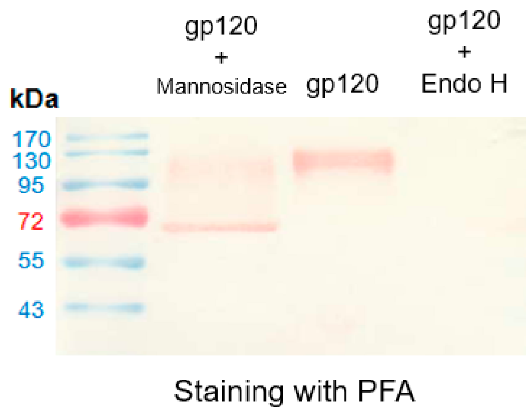

Figure S2. Additional experiment of PFA fusion probe staining against gp120 with and without glycosidase treatment. Western blot of gp120 that had undergone Mannosidase treatment (**left**), gp120 without glycosidase treatment (**middle**), and gp120 that had undergone Endo H treatment (**right**). The results were obtained after incubation of the membrane with the bound biotin mimetic peptide fusion PFA and strep-HRP (1:1 premix) for staining. No observed probe signal was seen for the right lane containing the Endo H treated gp120 as the glycans were removed. The middle lane reveals the approximate 120 kDa band for gp120 which was recognized by the PFA fusion probe. The left lane provides a weaker signal for the gp120 and at a slightly smaller molecular weight due to incubation with alpha-Mannosidase from jack bean resulting in cleavage of the terminal mannose residues from the gp120. This is expected as hybrid type glycans have been reportedly displayed by gp120 [1] that would be unaffected by the mannosidase and would thereby still provide the Man alpha(1-3)-Man alpha(1-6)-Man core to which the PFA has been reported to bind. The weaker signal would thus be representative of the reduction in the number of binding sites resulting from the Mannosidase cleavage of the Man alpha(1-3)-Man alpha(1-6)-Man core of high-mannose. In addition, a distinct band can be seen at the ~66 kDa size representative of the large subunit of the alpha-Mannosidase which from literature is known to possess a glucose-containing high-mannose-type glycan ($Glc_1Man_9GlcNAc_2$) and a small xylose- and fucose-containing complex-type glycan ($Xyl_1Man_1Fuc_1GlcNAc_2$) [2]. Note that the small 44 kDa subunit of the alpha-Mannosidase is not glycosylated and thus a band at 44 kDa could not be observed as there was no glycan for the PFA probe to bind.

1. Mizuochi, T.; Spellman, M.; Larkin, M.; Solomon, J.; Basa, L.; Feizi, T. Carbohydrate structures of the human-immunodeficiency-virus (HIV) recombinant envelope glycoprotein gp120 produced in Chinese-hamster ovary cells. *Biochem. J.* **1988**, *254*, 599–603.
2. Yoshinobu, K.; Hess, D.; Sturm, A. The N-glycans of jack bean alpha-mannosidase: Structure, topology and function. *Eur. J. Biochem.* **1999**, *264*, 168–175.
